# Scintillating Organic–Inorganic Layered Perovskite-type Compounds and the Gamma-ray Detection Capabilities

**DOI:** 10.1038/s41598-017-15268-x

**Published:** 2017-11-07

**Authors:** Naoki Kawano, Masanori Koshimizu, Go Okada, Yutaka Fujimoto, Noriaki Kawaguchi, Takayuki Yanagida, Keisuke Asai

**Affiliations:** 1 Graduate School of Materials Science, Nara Institute of Science and Technology, 8916-5 Takayama-cho, Ikoma, 630-0192 Japan; 2 Department of Applied Chemistry, Graduate School of Engineering, Tohoku University, 6-6-07 Aoba, Aramaki, Aoba–ku, Sendai, 980-8579 Japan

## Abstract

We investigated scintillation properties of organic–inorganic layered perovskite-type compounds under gamma-ray and X-ray irradiation. A crystal of the hybrid compounds with phenethyl amine (17 × 23 × 4 mm) was successfully fabricated by the poor-solvent diffusion method. The bulk sample showed superior scintillation properties with notably high light yield (14,000 photons per MeV) under gamma-rays and very fast decay time (11 ns). The light yield was about 1.4 time higher than that of common inorganic material (GSO:Ce) confirmed under ^137^Cs and ^57^Co gamma-rays. In fact, the scintillation light yield was the highest among the organic–inorganic hybrid scintillators. Moreover, it is suggested that the light yield of the crystal was proportional with the gamma-ray energy across 122–662 keV. In addition, the scintillation from the crystal had a lifetime of 11 ns which was much faster than that of GSO:Ce (48 ns) under X-ray irradiation. These results suggest that organic–inorganic layered perovskite-type compounds are promising scintillator for gamma-ray detection.

## Introduction

Scintillators are a type of phosphors. They immediately convert a single quantum of high energy (keV-GeV) radiation to a several thousands of low energy photons such as ultraviolet and visible light^1,2^, and they are used in various different fields such as nuclear medicine^3^, high-energy physics^4^, security^5^, and oil-dwelling^6^. The basic requirements for many applications are high light yield, fast decay, and high density, in addition to chemical stabilities and radiation hardness^1,2^. Time-of-flight positron emission tomography (TOF-PET) is an advanced technique of conventional PET, in which the image resolution can be improved by taking into account of travel distance of gamma-rays^7^. Therefore, it requires scintillators with considerably fast response. In general, the inorganic scintillators used in PET (e.g., GSO:Ce, LSO:Ce) have high density (7.0 g/cm^3^) and high light yield (over 10,000 photons/MeV), but they have long decay time (more than 40 ns)^1^. Another candidate is organic–inorganic hybrid scintillators (e.g., BC452) which exhibits fast decay (several ns) and high light yield (10,000 photons/MeV) but low density (1.0 g/cm^3^) and low chemical stability^8^. As in this example, scintillators with excellent time resolution and detection efficiencies are highly demanded.

Confined excitons in quantum structures are particularly interesting phenomenon which is potentially used for scintillators with faster decay and higher light yield. The binding energy of excitons in a two-dimensional system (2D, quantum well) is four times greater than that in the corresponding three-dimensional system (3D, bulk)^9,10^. In addition, the excitonic oscillator strength increases and excitonic radiative lifetime decreases with decreasing the dimension due to increasing an overlap between electron and hole wave-functions^11,12^. Owing to these quantum effects, excitons confined in quantum structures exhibit fast decay and high light yield. Therefore, confined excitions are a promising luminescence phenomenon applicable for a new class of scintillator materials.

We have been focusing on scintillation materials based on organic–inorganic layered perovskite-type compounds, (RNH_3_)_2_PbX_4_ (R: hydrocarbon group, X: halogen) among several compounds with quantum structures. The compounds have self-organized multiple quantum well structures with alternating organic–inorganic layers^13,14^. The inorganic layers of the quantum well consist of corner-sharing PbX_6_^2−^ octahedra that are sandwiched between the organic barrier layers. Excitons in the inorganic layer possess large oscillator strength and exciton binding energy due to the quantum confinement effect and image-charge effect^15,16^. In addition to the unique optical properties such as electroluminescence^17^ and distinguished optical nonlinearities^18^, scintillation properties of the hybrid compounds have been investigated under various types of radiation. Efficient scintillation owing to the exciton recombination in the inorganic layer was observed under proton, electron, and X-ray irradiations^19–21^.

Such optical properties under optical and ionizing irradiations are governed by exciton properties in the inorganic layer. In our previous studies, we have investigated the correlation between electronic structure in the inorganic layer and optical properties under various types of radiation^22–24^. Based on structure analysis and photoluminescence spectroscopy, it has been demonstrated that luminescence properties of the hybrid compounds are governed by structural distortions in the Pb–Br–Pb bondings between the adjoining PbBr_6_^2−^ octahedra and Br–Pb–Br bond inside the PbBr_6_^2−^ octahedra in the inorganic layer^22^. In addition to the luminescence properties, it has been shown that scintillation properties are also governed by the exciton properties in the inorganic layer under synchrotron X-ray irradiation (67.4 keV), because the effect of the energy transfer from an organic to an inorganic layer on the scintillation properties are negligible owing to much lower energy deposited in the organic layer than that in the inorganic layer^23,24^. Therefore, (C_6_H_5_C_2_H_4_NH_3_)_2_PbBr_4_, which has both the distortions in the inorganic layer, is promising candidate as a gamma-ray scintillator material.

In this study, we investigated photoluminescence (PL) and scintillation properties of organic–inorganic layered perovskite-type compounds under gamma-ray and X-ray irradiations. A hybrid compound crystal (phenethyl amine incorporated into the organic layer) was fabricated by the poor solvent diffusion method. Further, the scintillation spectra, scintillation decay profiles and pulse-height spectra of the (C_6_H_5_C_2_H_4_NH_3_)_2_PbBr_4_ (or Phe) crystal were characterized.

## Results

**Figure 1:**
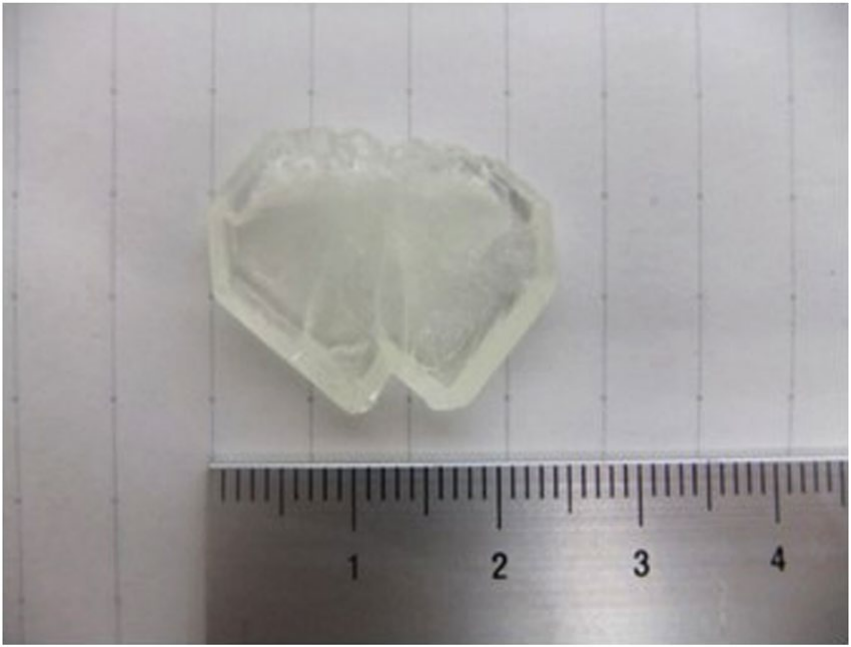
Photograph of the crystal of Phe crystal.

Figure 1 illustrates a photograph of a prepared Phe crystal. The size of the crystal was 17 × 23 × 4 mm^3^ approximately. The transparency was not so high that the line patterns on the back of the sample were not seen clearly. The visual observations indicated that some cracks and defects such as grain boundaries were included.

**Figure 2:**
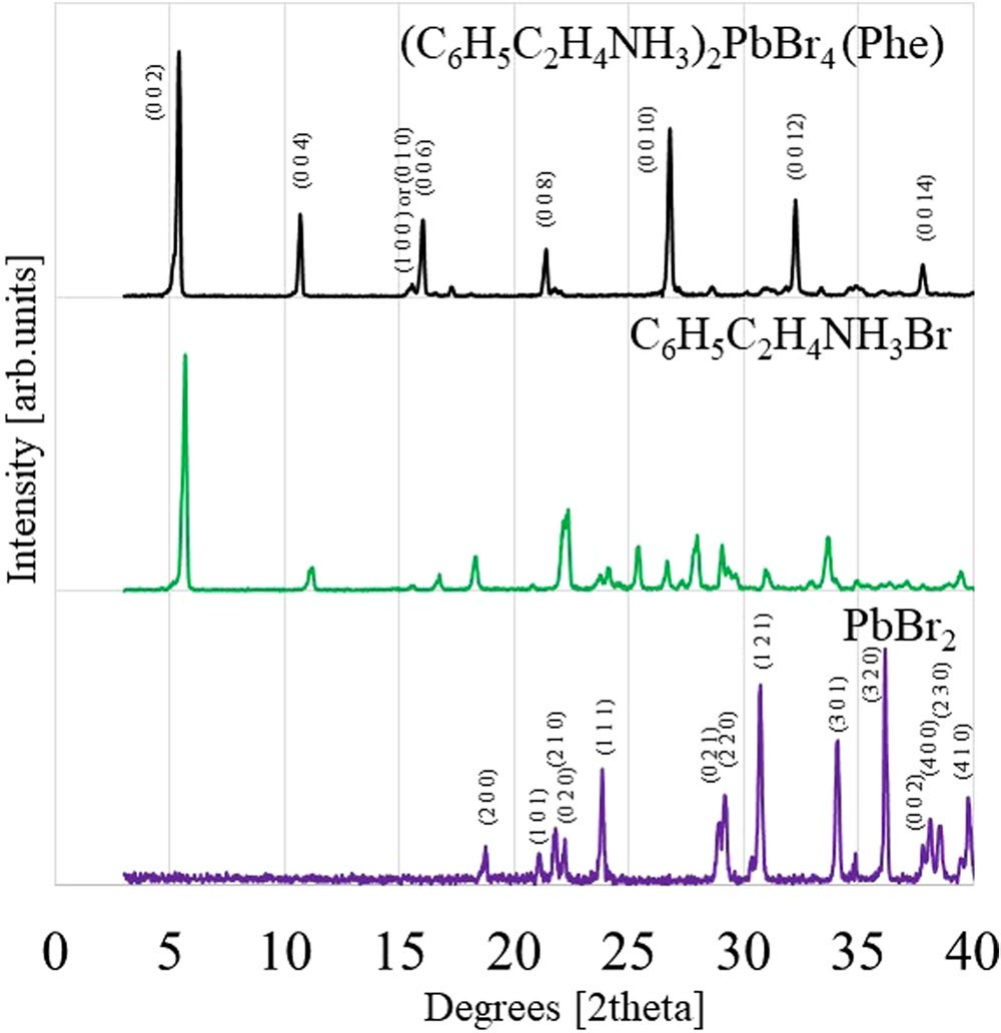
X-ray diffraction patterns of Phe, C_6_H_5_C_2_H_4_NH_3_Br and PbBr_2_.

Figure 2 shows the XRD patterns of the Phe crystal, C_6_H_5_C_2_H_4_NH_3_Br and PbBr_2_. Some of the diffraction peaks of the Phe crystal and all the diffraction peaks of PbBr_2_ were identified while the diffraction peaks of C_6_H_5_C_2_H_4_NH_3_Br could not be identified due to the absence of crystalline data in the database. The observation of (0 0 2 *l*) diffraction patterns of the Phe crystal, where *l* = 1–7, explains that two dimensional quantum structure was formed in the Phe crystal. The lattice constant of c-axis was estimated to be about 16.4 Å. Each the (0 0 2 *l*) diffraction peak was a single peak, and no peak separation was observed. Hence, the obtained Phe crystal had no phase separation. However, a small amount of undesirable impurities except for the precursors such as C_6_H_5_C_2_H_4_NH_3_Br and PbBr_2_ may be included in the Phe crystal.

**Figure 3:**
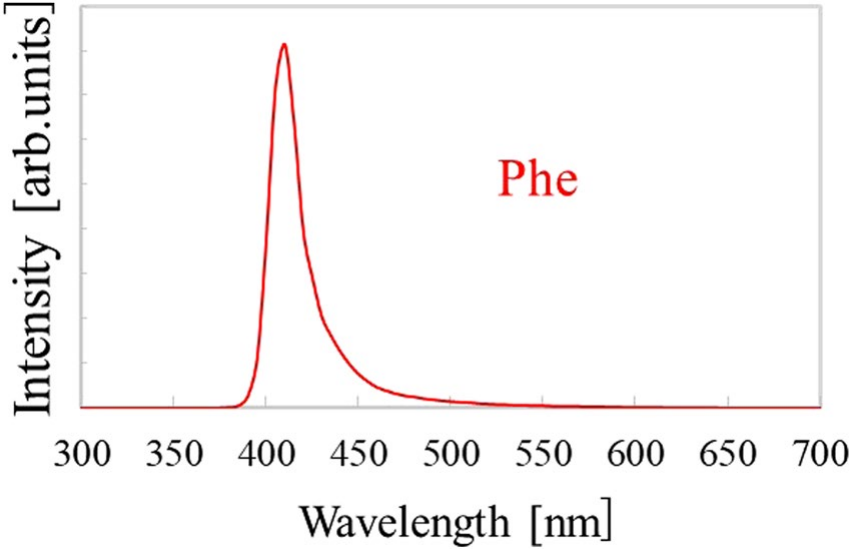
PL spectra of Phe under excitation of 280 nm.

Figure 3 exhibits PL spectra of the Phe crystal under excitation of 280 nm. A PL peak was observed at 410 nm in the Phe crystal. The emission wavelength of the Phe crystal agreed well with the reported value for Phe spin-coated film^25^. Therefore, the PL peak can be ascribed to exciton emissions from the inorganic layer^22,25^. According to ref.^25^, the Stokes shift in the Phe spin-coated film was less than 10 meV, so self-absorption and reemission of excitons can occur in the Phe crystal. Moreover, quantum efficiency of the Phe crystal under excitation of 300 nm was 0.25, which was almost equivalent to the value of Phe single crystal reported in our previous study^22^.

**Figure 4:**
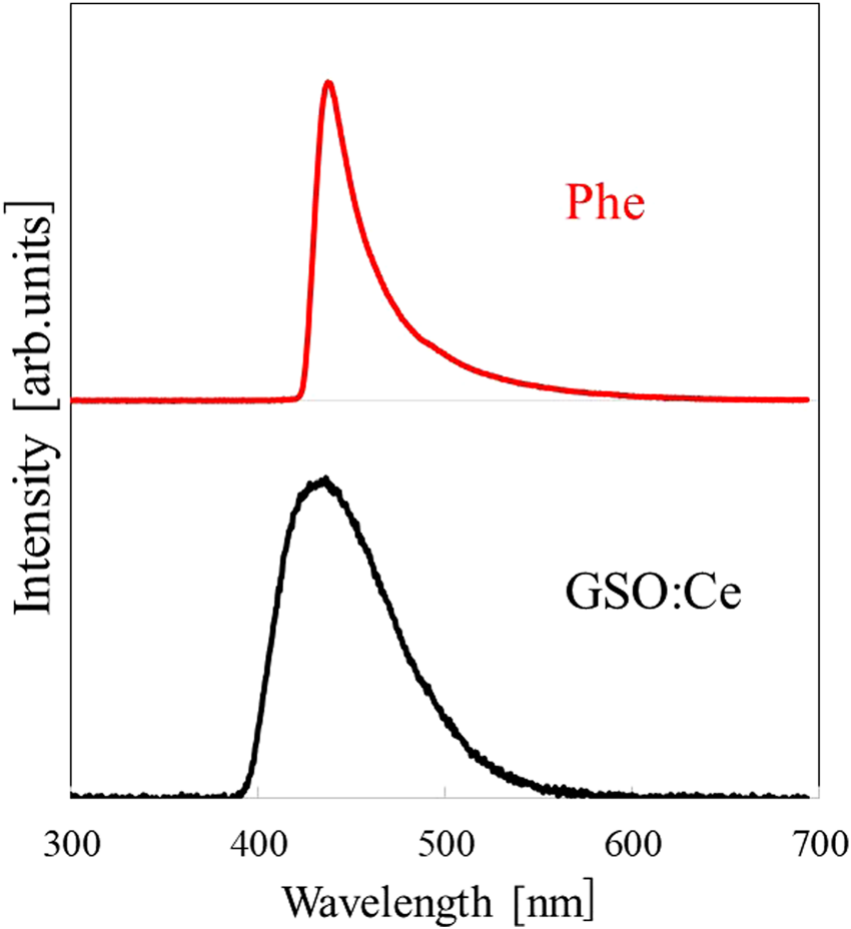
Scintillation spectra of Phe and GSO:Ce under X-ray irradiation.

Figure 4 exhibits X-ray induced scintillation spectra of the Phe crystal and GSO:Ce as a reference. A scintillation peak at 437 nm was observed for the Phe crystal. This sharp peak was attributed to exciton emission from the inorganic layer according to the PL spectrum (Fig. 3) and the previous studies^21,26^. In addition, the emission from GSO:Ce at 434 nm was due to the 5d-4f transitions of Ce^3+^ as typical and reported earlier^27,28^.

**Figure 5:**
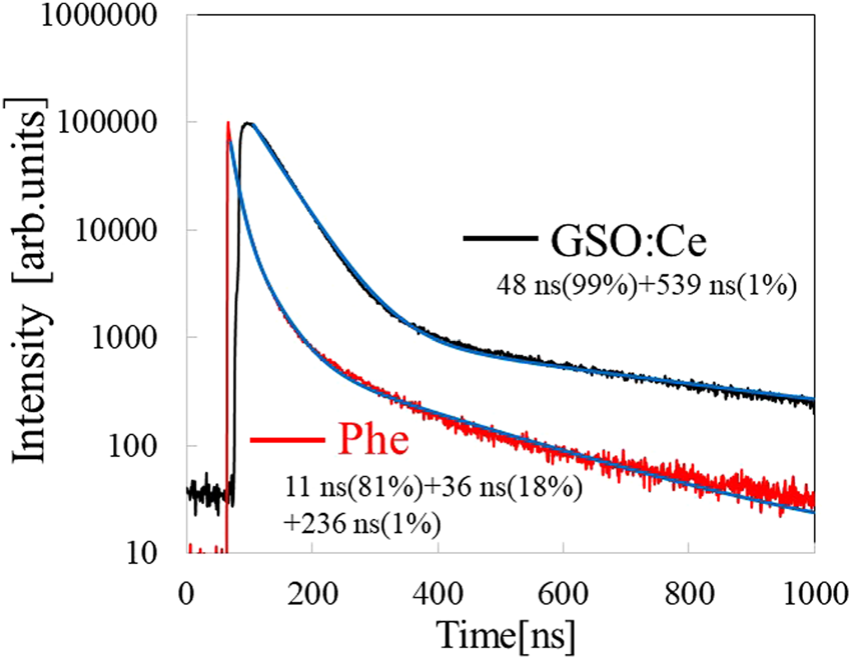
Scintillation decay time profile of Phe and GSO:Ce under X-ray irradiation.

Figure 5 represents scintillation decay profile of the Phe crystal and GSO:Ce measured by X-ray irradiation. The profile was fitted with multi-exponential decay curves. The approximation led components of three different lifetimes: 11 ns (81%), 36 ns (18%), and 236 ns (1%). The first component can be attributed to the recombination of excitons in the inorganic layer, based on our previous studies^24^. In addition, the lifetime (11 ns) was much faster than that of GSO:Ce (48 ns) in which the emission is due to the 5d-4f transitions of Ce^3+^. Therefore, the Phe crystal exhibits greater scintillation properties–significantly shorter decay in addition to higher light yield compared with GSO:Ce.

**Figure 6:**
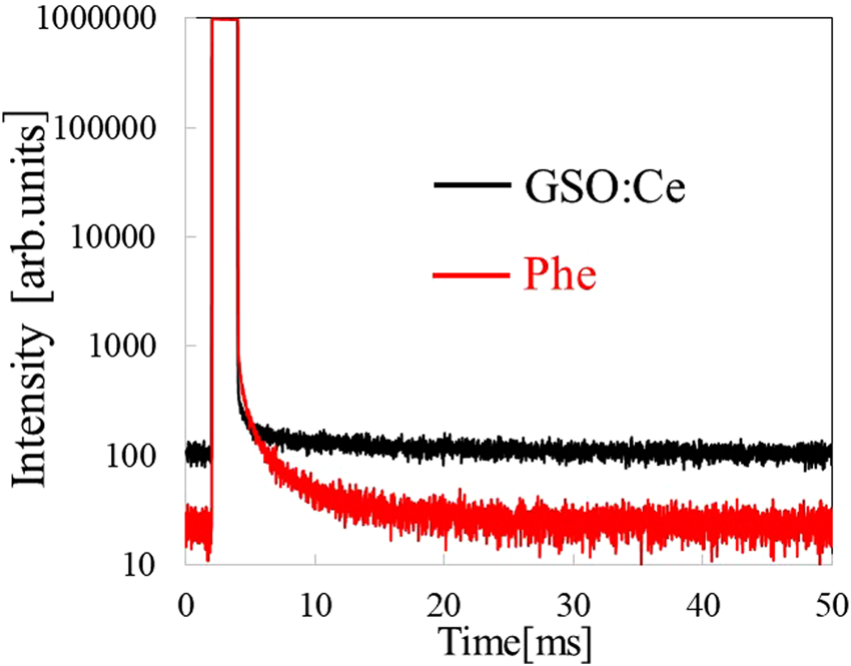
X-ray induced afterglow time profiles of Phe and GSO:Ce.

Figure 6 compares X-ray induced afterglow profiles of the Phe crystal and GSO:Ce. The afterglow level (*A*) was defined as *A* (%) = 100 × (*I*_2_−*I*_BG_)/(*I*_1_−*I*_BG_) where *I*_BG_ is the background signal, *I*_1_ is the averaged signal intensity during X-ray irradiation and *I*_2_ is the signal intensity at 20 ms after X-ray irradiation. The afterglow levels of the Phe crystal and GSO:Ce were 5 ppm and 15 ppm, respectively. The afterglow level of the Phe crystal was almost equivalent to those of commercial scintillators CdWO_4_ and BGO^29^. According to ref.^30^, activation energies of trapping sites in organic-inorganic layered perovskite-type compounds are very shallow^30^. The compounds are formed in a self-organized manner, so the creation of lattice defects and lattice mismatch between the organic and inorganic layers is inhibited. This should be the reason why the afterglow level of the Phe crystal was very low.

**Figure 7:**
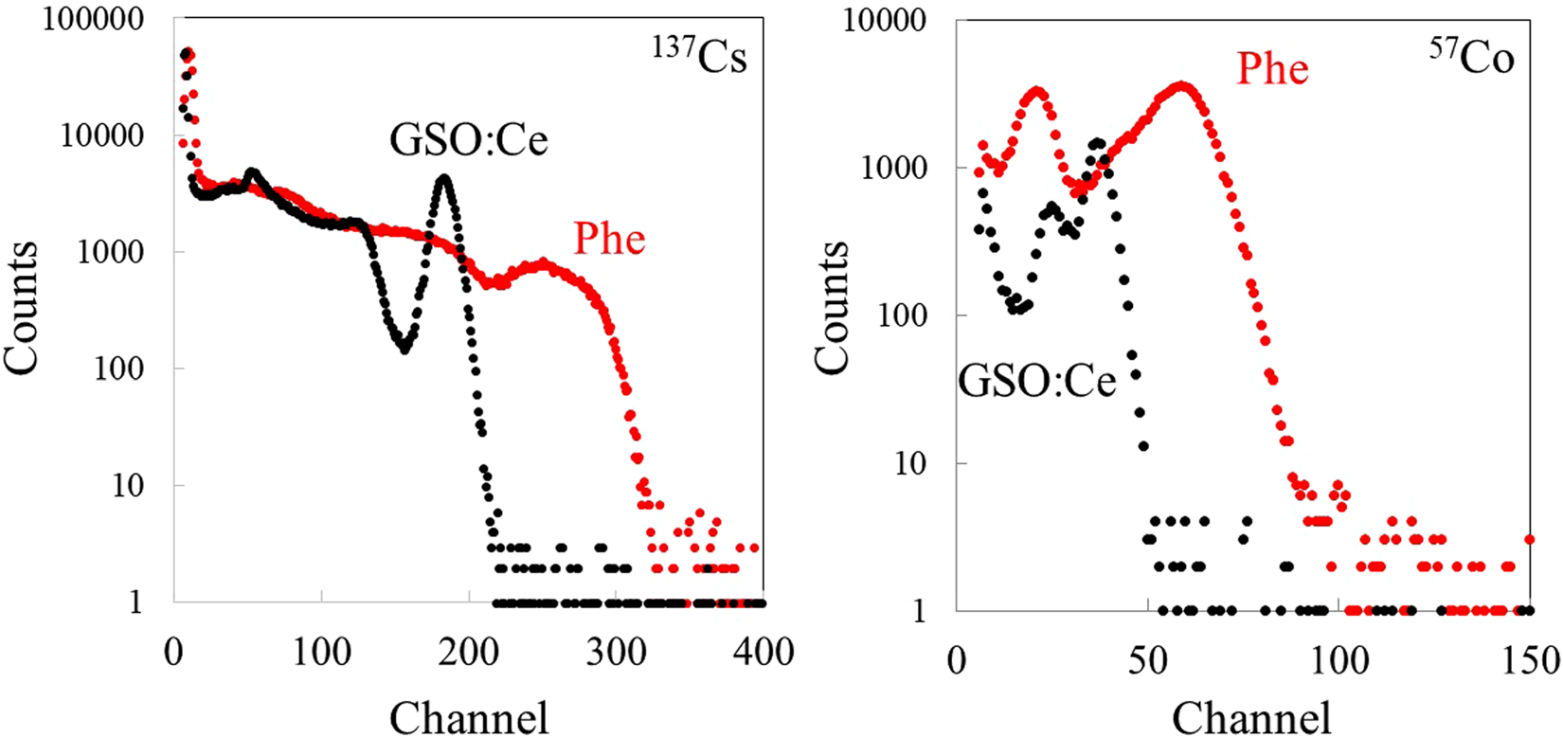
^137^Cs and ^57^Co induced pulse height spectra of Phe and GSO:Ce.

Figure 7 shows pulse-height spectra measured using the Phe crystal and those using GSO:Ce as a comparison. The gamma-ray sources used are ^137^Cs (662 keV) and ^57^Co (122 keV). The pulse height channel of Phe and GSO:Ce was 247 ± 10 (Phe, ^137^Cs), 107 ± 5 (Phe, ^57^Co), 182 ± 5 (GSO:Ce, ^137^Cs), 77 ± 3 (GSO:Ce, ^57^Co). The scintillation light yield of the Phe crystal was about 1.4 times higher than that of GSO:Ce under gamma-ray irradiation of both ^137^Cs and ^57^Co, on an assumption that the pulse height was proportional to the scintillation output. In our previous studies, the scintillation light yield of GSO:Ce was evaluated using reverse-type avalanche photodiodes (APDs) as scintillation detectors^31^. According to the evaluation system calibrated by using ^55^Fe (5.9 keV), the scintillation light yield of GSO:Ce was estimated to be 10,000 photons/MeV. Based on the registered channel number and the light yield of GSO:Ce, the scintillation light yield of the Phe crystal was estimated to be 14,000 photons per MeV. Regarding the energy resolution ΔE(FWHM)/E, the Phe crystal showed 29 ± 6%(^137^Cs) and 43 ± 7%(^57^Co) and that of GSO:Ce was 9 ± 2%(^137^Cs) and 19 ± 2%(^57^Co), which was approximately consistent with those reported by the previous studies^32,33^. The poor energy resolution of the Phe crystal can be ascribed to crystal nonuniformity and self-absorption of excitons in the inorganic layer.

**Figure 8:**
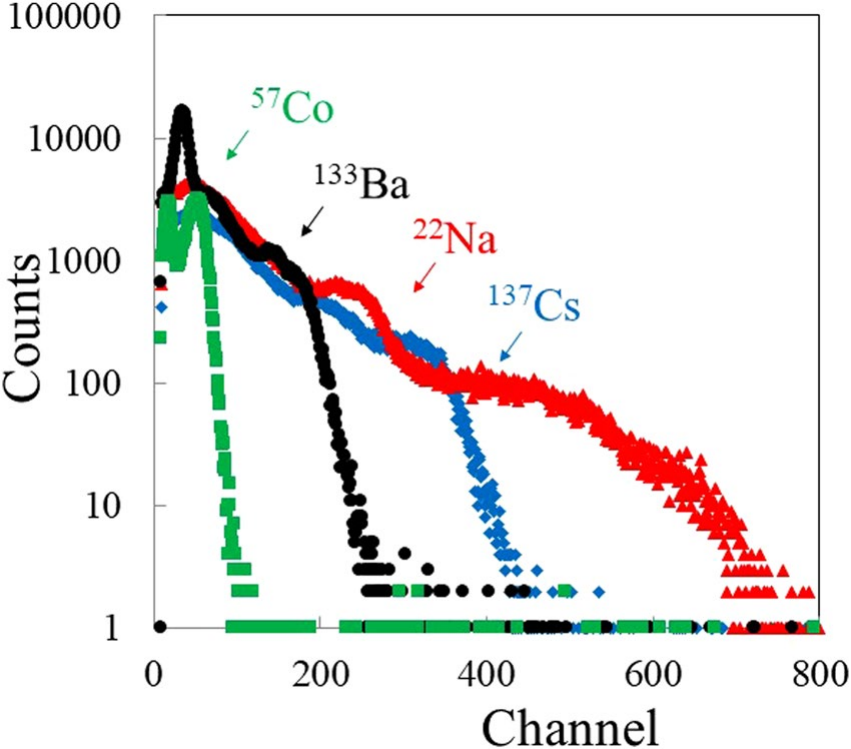
Pulse height spectra of Phe under various gamma-ray sources.

**Figure 9:**
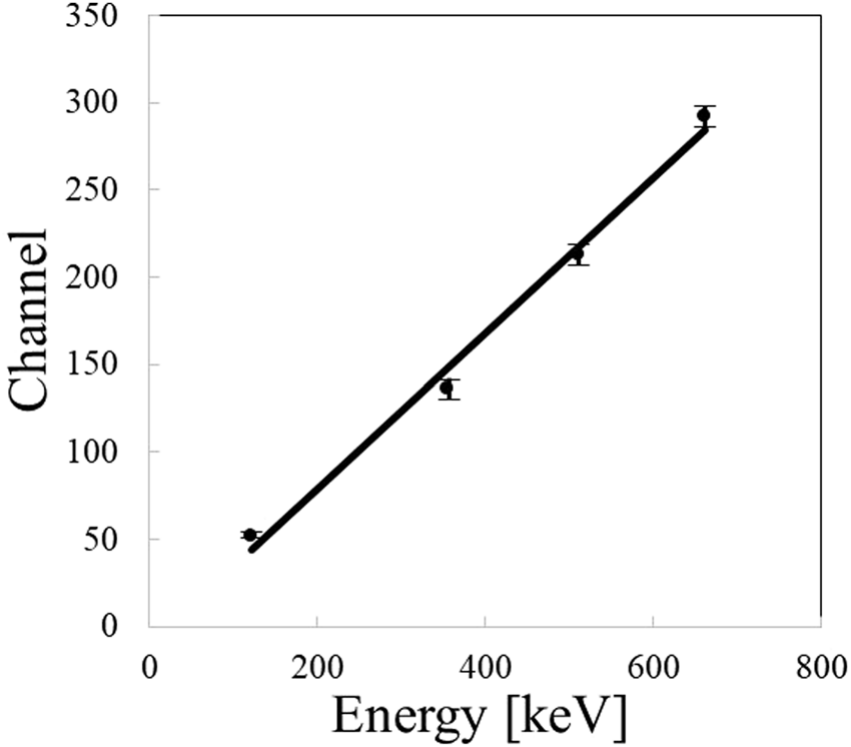
Correlation between gamma-ray energy and pulse-height channel.

Figure 8 exhibits pulse-height spectra of ^57^Co (122 keV), ^133^Ba (356 keV), ^22^Na (511 keV), and ^137^Cs (662 keV) gamma-ray sources measured by using the Phe crystal sample. Pulse height peaks were successfully observed under each gamma-ray sources. The registered channel was 52 ± 2 (^57^Co), 136 ± 5 (^133^Ba), 213 ± 5 (^22^Na), and 292 ± 5 (^137^Cs). In addition, the energy resolution ΔE(FWHM)/E of the Phe crystal under each gamma-ray sources was measured to be 49 ± 6% (^57^Co), 58 ± 7% (^133^Ba), 55 ± 5% (^22^Na), and 35 ± 5%(^137^Cs), respectively. Figure 9 represents the correlation between gamma-ray energy and the corresponding pulse height channel. It is suggested that the pulse height channel (in turn scintillation light yield) is proportional to the gamma-ray energy. These results suggest that organic–inorganic layered perovskite-type compounds are potential materials for determination of the energy of the detected radiation in the 122–662 keV energy range.

## Discussion

According to Figs 5 and 7, the Phe crystal exhibited significantly higher scintillation light yield and faster decay compared with those of GSO:Ce. In our previous study, the effect of organic moieties on the scintillation properties of organic–inorganic layered perovskite-type compounds has been investigated. In case of scintillation, both an organic and an inorganic layers are excited because the excited energy of benzene is about 4.7 eV^34^. Recently, we demonstrated that scintillation properties of organic–inorganic layered perovskite-type compounds mainly depend on exciton properties of the inorganic layer because deposited energy of the inorganic layer is much higher than that of the organic layer when the compounds are irradiated by gamma-rays or X-rays. Therefore, significant scintillation shown in Figs 5 and 7 can be achieved by the excitons confined in the quantum well layer (the inorganic layer). In addition, scintillation light yield of the Phe crystal exhibits 14,000 photons per MeV, which was higher than that of GSO:Ce (10, 000 photons per MeV) and commercial organic–inorganic hybrid scintillators such as BC-452. Our structural analyses and photoluminescence spectroscopy suggested that structural distortion in the inorganic layer affects the intensity of luminescence, because these distortions lead to a decrease in the Bohr radius of the excitons^35,36^. This is the reason why the Phe crystal, which has both distortions in the adjoining PbBr_6_^2−^ octrahedra and inside the PbBr_6_^2−^ octahedron, exhibited a high scintillation light yield.

In addition, it is suggested that scintillation light yield of the Phe crystal was proportional to the gamma-ray energy as illustrated in Fig. 9. The correlation between gamma-ray energy and scintillation light yield for GSO:Ce has been investigated experimentally and theoretically^33,37^. Scintillation light yield of GSO:Ce was non-proportional to the gamma-ray in the 10–1000 keV energy range, due to the effects of K- and L-edge absorptions and non-radioactive processes^33,37^. On the other hand, it is suggested that scintillation light yield of the Phe crystal was proportional to the gamma-ray energy in the 122–662 keV range. The proportionality can be attributed to the following reasons. The K-edge absorption energy of Pb was about 85 keV, which was out of the range of 122–662 keV^38,39^. In addition, organic–inorganic layered perovskite-type compounds are formed in a self-organized multiple quantum structure. Therefore, size distributions and creation of lattice defects related to non-radiative processes are avoided. Further investigations of the correlation between gamma-ray energy and scintillation light yield should be required due to the poor energy resolution. These results suggest that organic–inorganic layered perovskite-type compounds are promising scintillator material for gamma-ray detections.

## Methods

### Synthesis

Stoichiometric quantities of phenethylamine (C_6_H_5_C_2_H_4_NH_2_) and hydrobromic acid were reacted in water for 0.5 h. After evaporation of the solvent, C_6_H_5_C_2_H_4_NH_3_Br powder was obtained and subsequently dissolved in N,N-dimethylformamide (DMF) with PbBr_2_ at a molar ratio of 2:1 and then stirred for 3 h under dry argon flow. Powder of (C_6_H_5_C_2_H_4_NH_3_)_2_PbBr_4_ was then obtained by evaporating the solvent. Furthermore, the obtained powder was processed by the poor-solvent diffusion method in order to fabricate into a single crystal of (C_6_H_5_C_2_H_4_NH_3_)_2_PbBr_4_ as follows. The obtained (C_6_H_5_C_2_H_4_NH_3_)_2_PbBr_4_ were dissolved in DMF, as a strong solvent, in a glass bottle (50 ml), and then nitromethane, as a poor solvent, was dropped into the solution until just before precipitation. Next, the bottle was loaded in a shaded desiccator where the poor solvent was poured at the bottom. The vapor of the poor solvent was gradually diffused into the solution to reduce the solubility. It took for a month to obtain single crystals of Phe, which were grown in the bottom of the bottles. Furthermore, a piece of the obtained single crystals was used as a seed crystal and loaded into the new bottle including Phe powder, DMF, and nitromethane solution. Over another month of crystal growth, a larger size of the Phe crystal was obtained. By repeating the above procedures several times, a crystal with a thickness of 4 mm was obtained.

### Evaluation of the sample

The crystal structure was investigated by X-ray diffraction (XRD) over a 2θ range of 3° to 40° at room temperature using Cu Kα radiation. The quantum efficiency was measured by using Quantaurus QY (C11347, Hamamatsu). The PL decay curve was measured by using Quantaurus τ (C11367, Hamamatsu). In these measurements, the excitation wavelength was 280 nm which was the shortest excitation wavelength available in the instrument, and the monitoring wavelength was 410 nm. X-ray induced scintillation spectra were measured by our original setup^40^. A conventional X-ray tube which is equipped with a W anode target (XRB80P & N200 × 4550, Spellman) and a Be window was used as an excitation source. During the operation, the tube voltage and current was set to 40 kV and 5.2 mA, respectively. The scintillation photons from the sample were led to a spectrometer (an assembly of Andor DU-420-BU2 CCD and Shamrock 163 monochromator) through a 2.0 m optical fiber. Here, the spectrometer was placed off the irradiation geometry axis to avoid X-ray photons directly striking onto the CCD. In order to reduce the thermal noise, the CCD element was cooled down to 193 K by a Peltier module. Pulse height spectrum measurements were performed to estimate scintillation light yields. A crystal sample was placed on a window of photomultiplier tube (PMT; R7600-2000, Hamamatsu) with an optical grease. The sample was covered with several layers of Teflon tape to guide all the scintillation photons towards the PMT. The high voltage of −700 V was supplied (ORTEC 556), and the signals were read out from the anode of the PMT. In order to separate from the background gamma-rays, the detector assembly (PMT with scintillator sample) was placed inside Pb walls with a thickness of 5 cm. Once a gamma-ray was detected, the signals were fed into the pre-amplifer (ORTEC 113) and then to the shaping amplifer (ORTEC 572) with 1 µs shaping time. After converting to digital signals by a multi channel analyzer (Amptek, Pocket MCA 8000 A), they were recorded to a computer. The X-ray induced scintillation decay profiles and the X-ray induced afterglow profiles were measured using an afterglow characterization system^29^, which was equipped with a pulsed X-ray tube. The repetition frequency was 200 kHz for scintillation decay measurements and 10 Hz for afterglow measurements. The X-ray source was supplied with the voltage of 30 kV during the measurement. The systems integrate the emission signal over the wavelength range of approximately 160–650 nm. A commercial GSO:Ce scintillator was used as a standard in order to compare the scintillation properties.
